# IgG light chain-independent secretion of heavy chain dimers: consequence for therapeutic antibody production and design

**DOI:** 10.1042/BCJ20170342

**Published:** 2017-09-08

**Authors:** Chloe L. Stoyle, Paul E. Stephens, David P. Humphreys, Sam Heywood, Katharine Cain, Neil J. Bulleid

**Affiliations:** 1 Institute of Molecular, Cell and Systems Biology, CMVLS, University of Glasgow, Davidson Building, Glasgow G12 8QQ, U.K.; 2 UCB Pharma Slough, 208 Bath Road, Slough SL1 3WE, U.K.

**Keywords:** antibodies, antibody folding, antibody production, antibody secretion, endoplasmic reticulum

## Abstract

Rodent monoclonal antibodies with specificity towards important biological targets are developed for therapeutic use by a process of humanisation. This process involves the creation of molecules, which retain the specificity of the rodent antibody but contain predominantly human coding sequence. Here, we show that some humanised heavy chains (HCs) can fold, form dimers and be secreted even in the absence of a light chain (LC). Quality control of recombinant antibody assembly *in vivo* is thought to rely upon folding of the HC C_H_1 domain. This domain acts as a switch for secretion, only folding upon interaction with the LC C_L_ domain. We show that the secreted heavy-chain dimers contain folded C_H_1 domains and contribute to the heterogeneity of antibody species secreted during the expression of therapeutic antibodies. This subversion of the normal quality control process is dependent on the HC variable domain, is prevalent with engineered antibodies and can occur when only the Fab fragments are expressed. This discovery will have an impact on the efficient production of both humanised antibodies and the design of novel antibody formats.

## Introduction

The ability to humanise rodent antibodies efficiently along with improvements in recombinant expression has led to the development of several high-value and effective therapeutics [1]. However, efforts to produce novel antibody formats such as bi- or tri-specific molecules have met with difficulties in terms of low titres and poor yields, resulting in longer production runs and the requirement for extensive purification [2]. The heterogeneity in expressed protein is likely to be caused by a lack of efficient folding and assembly of the desired antibody and the subsequent secretion of alternate assemblies. Given the potential for antibodies and their derivatives as therapeutics, any improvements in optimising the production process in terms of yield and quality will have an impact on their utility and affordability.

The correct folding and assembly of antibodies requires an elegant cellular quality control process [3]. Crucially, heavy chains (HCs) are retained in the cell as dimeric intermediates until two light chains (LCs) assemble with the HC dimer to form a tetramer, which is then released from this retention and secreted from the cell [4]. The HC dimer is retained due to a lack of folding of the C_H_1 domain in the absence of the cognate LC, resulting in a high-affinity interaction with the intracellular chaperone, immunoglobulin HC-binding protein (BiP) [5,6]. This interaction is reversed upon LC assembly with the C_L_ domain acting as a template to allow correct folding of the C_H_1 domain [7,8]. This cellular mechanism ensures that only correctly folded and assembled molecules are secreted from an antibody-producing cell. The quality control system also functions in Chinese hamster ovary (CHO) cells used for commercial antibody production with BiP retaining unassembled HC dimers [9]. However, the fidelity of this retention has not been analysed for engineered antibodies as it has been assumed that the retention is efficient, at least during the expression of commonly expressed IgG formats.

While the retention mechanism seems to hold for many natural antibodies, HC dimers can be secreted in LC-deficient mice if they lack the C_H_1 domain [6,10]. Camelids also express HCs that lack a C_H_1 domain, allowing the secretion of functional HC dimers [11]. In addition, some transcripts found in mammalian pro-B-cells encode HCs that can be expressed on the cell surface in the absence of any surrogate or conventional LC [12]. These HCs contain a C_H_1 domain, which folds in the absence of a LC, enabling them to escape the endoplasmic reticulum (ER) quality control mechanism. Interestingly, these HCs are not found in pre- or mature B-cells, indicating the presence of a negative selection for cells expressing HCs that can be secreted in the absence of a LC. Importantly, these HCs differ only in their variable domain, indicating that the requirement for LC-dependent folding of the C_H_1 domain is an intrinsic property of the V_H_ domain that is selected for during B-cell development.

In the present study, we show that some engineered HCs can also be secreted as dimers. The C_H_1 domain within these HC constructs can fold in the absence of the cognate LC, thereby subverting the cellular quality control process. The results suggest that the humanisation process can inadvertently create HC constructs that would normally be selected against during B-cell development.

## Experimental procedures

### Cell lines and DNA constructs

CHO adherent cells (CHO) L761H were grown in Dulbecco's Modified Eagle Medium (DMEM) containing 10% foetal calf serum. CHO-S suspension cells (Life Technologies) were grown in CD CHO chemically defined medium. All HCs contained either the human IgG1 or IgG4 C_H_1,2,3 domains fused to either mouse or humanised V_H_ domains with specificity for A33 [13], TNF-α [14], CD20 [15], CD25 (Zenapax) [16], CD25 (Simulect) [17] and CD11a [18]. The variable domain from an in-house humanised mouse antibody (277) was also used [19]. Variable domains were synthesised by DNA 2.0. All IgG4 HCs contain an S225P mutation (i.e. –ESKYGPPCPSCP– to –ESKYGPPCPPCP–), which reduces the amount of HC : LC dimers formed [20]. Additional mutations introduced by site-directed mutagenesis included C127S in the A33 Fab fragment C_H_1 domain and P151A in the A33 HC C_H_1 domain as indicated. The Fab HC constructs were prepared by PCR using primers complementary to the 5′ sequence and to the junction between the C_H_1 domain and the hinge region. The 3′-primer was also designed to add a V5-tag and a stop codon. The final construct is depicted in Figure 4B.

### Transient transfections

CHO-S cells were transfected for western blot analysis, and CHO-L761H were transfected for the pulse labelling experiments. The transfection reagent used was either polyethylenimine (PEI) [21] or NovaCHOice [22] (Novagen) as indicated. For PEI transfections, the ratio of DNA : PEI was 1 : 2.5. DNA was added to serum-free medium (CHO-L761H) or CD CHO (CHO-S) and incubated for 5 min. PEI was then added, mixed and incubated for a further 10 min. The DNA and PEI mixture was added to cells in complete medium or CD CHO medium. After 24 h, the medium was removed and replaced with complete medium or CD CHO. The transfected cells and medium were analysed 48 h post transfection.

For NovoCHOice transfections, 20 µg of DNA and 20 µl of reagent were mixed in serum-free medium or CD CHO and incubated for 10 min. The mixture was then added to cells in complete medium or CD CHO and incubated for 48 h prior to analysis.

### Western blot analysis

Transfected cells and medium were separated by centrifugation. *N*-ethyl maleimide (NEM) was added to the medium to a final concentration of 20 mM, and cells were incubated in phosphate-buffered saline containing NEM (20 mM) for 10 min to trap the disulphide status of secreted or intracellular proteins. Cell lysates were prepared using lysis buffer [50 mM Tris–HCl buffer (pH 7.5), containing 150 mM NaCl, 5 mM EDTA, 1% (v/v) Triton X-100 and 20 mM NEM]. Cell lysates or medium was centrifuged at 9500×***g*** for 10 min to pellet the cell debris. The supernatant from the cell lysis or the medium was pre-cleared using 10% Sepharose beads for 30 min before affinity purification with 1% protein A-Sepharose beads overnight. Isolation of the A33 Fab product containing a V5-epitope tag from medium or cell lysate was carried out using anti-V5-agarose affinity gel beads (Sigma). Protein A or V5-agarose beads were washed with buffer A [50 mM Tris–HCl (pH 8), 1% Triton X-100, 150 mM NaCl, 2 mM EDTA, 0.5 mM phenylmethylsulfonyl fluoride] before adding SDS–PAGE sample buffer [50 mM Tris–HCl (pH 6.8), containing 2% (w/v) SDS, 0.1% (w/v) bromophenol blue and 10% (v/v) glycerol]. Samples were reduced with 10 mM dithiothrietol (DTT) where indicated prior to gel electrophoresis. Proteins were separated by SDS–PAGE on gradient (4–20%), 7.5% or 12.5% polyacrylamide gel as indicated. The proteins were transferred to nitrocellulose membranes (Li-COR). Membranes were treated for 1 h at room temperature in 5% (w/v) milk and incubated with a fluorescent protein A [23] or mouse anti-V5 (1/10 000) (Invitrogen: R96025) or mouse anti-human IgG HC (1/1000) (Abcam: AB7500) for 1 h. If a primary antibody was used, then the membrane was developed using fluorescent protein A. Fluorescent protein was detected using an Odyssey Li-COR Sa imaging system.

### Pulse-chase assay

Transfected CHO-L761H cells for pulse labelling were starved in minus cysteine and minus methionine (−cys−met) DMEM for 30 min. The medium was replaced with fresh –cys−met with the addition of a radiolabelled methionine and cysteine mixture (^35^S) for 30 min at a concentration of 110 µCi/ml. Cells were washed in PBS and subsequently incubated in complete DMEM containing 0.5 mM cycloheximide for varying chase times as indicated. Cell lysates were prepared as described above and protein samples were immunoisolated using mouse anti-κ-LC (1/1000) (Sigma: K4377) and mouse anti-human IgG antibodies and incubated overnight with 1% protein A-Sepharose. The beads were washed with buffer A and eluted in SDS–PAGE sample buffer. Protein samples were separated by SDS–PAGE on a gradient (4–20%) polyacrylamide gel. The gel was fixed using a solution of 10% (v/v) methanol and 10% (v/v) acetic acid for 20 min, and then dried prior to exposure to a phosphorimager plate and developed on a Fuji FLA-7000 phosphorimager.

### 2D Gel electrophoresis

A pulse-chase assay was carried out as described with a chase time of 3 h. The medium and cells were treated with NEM and cell lysate, and medium was prepared as described above. Antibody chains were isolated with anti-κ-LC and anti-HC-IgG antibodies along with protein A-Sepharose as described above. A 4–20% gradient gel was run as the first dimension. A sample lane was then cut (∼3 mm wide) and incubated in 50 mM DTT for 10 min to reduce the proteins in the gel. An identical sample lane was fixed in 10% (v/v) acetic acid/10% (v/v) methanol. The gel lane incubated in 50 mM DTT was placed horizontally along the top of a second, thicker gel (1.5 mm, 12.5%) and sealed with 1% (w/v) agarose. The gel was then run at 20 mA and fixed in 10% (v/v) acetic acid/10% (v/v) methanol. The gel along with the fixed sample lane from the first dimension was dried and exposed to a phosphorimage plate overnight.

### Endoglucosidase H and peptide : *N*-glycosidase F treatment

CHO-S cells transfected with A33 HC IgG4 were split into three samples, and the medium and cells were separated by centrifugation. Cell lysates and medium were prepared and HCs were purified using protein A-Sepharose as described above. Protein A-Sepharose beads were washed three times with buffer A and then resuspended in 50 µl of denaturing buffer (0.5% SDS and 0.04 M DTT). Each sample was boiled for 10 min and centrifuged at 16 000×***g*** for 1 min. Samples were treated with endoglycosidase H (1000 U, New England BioLabs) or peptide : *N*-glycosidase (PNGase F) (1000 U, New England BioLabs) in 50 mM sodium citrate buffer (pH 5.5) or 50 mM sodium phosphate buffer (pH 7.5), respectively [24]. All samples were incubated at 37°C overnight. Samples were separated by SDS–PAGE on a gradient (4–20%) polyacrylamide gel. The gel was silver-stained to visualise the proteins.

### Size-exclusion HPLC

Samples for size-exclusion (SE) chromatography were purified from culture medium from transfected CHO-S cells using protein A-Sepharose. Samples (∼20 µg) were loaded onto a TSKgel G3000SW, 10 µm, 7.5 mm ID × 300 mm column (Tosoh) and developed with an isocratic gradient of 0.2 M sodium phosphate (pH 7.0) at 1 ml/min for 17 min. Detection was by absorbance at 280 nm. Apparent molecular masses were calculated using the sample peak retention time and the interpolated retention times for Bio-Rad gel filtration markers (Cat. No. 151-1901)*.*

## Results

### IgG LC-independent secretion of HCs

**Figure 1.**
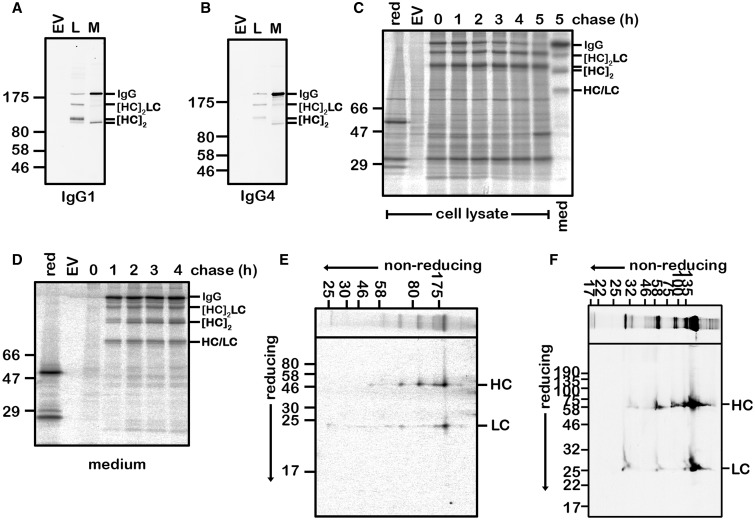
HC dimer secretion in the presence of LC. CHO-S (**A** and **B**) or CHO-L761H (**C**–**E**) cells were co-transfected with expression constructs coding for the IgG1 (**A**) or IgG4 (**B**–**E**) A33 HC and LC. Cells transfected with the empty vector are as indicated (EV). (**A** and **B**) Immunoblot analysis of the intracellular (L) and secreted (M) antibody products separated on a 10% polyacrylamide gel. Fluorescent protein A was used to detect antibody products containing the Fc dimer of the HC. The identity of the products are as indicated. (**C** and **D**) Time course of expression and secretion of the various antibody intermediates present in the lysate (**C**) or medium (**D**) determined by pulse-chase analysis. The antibody species were immunoisolated using anti-LC and anti-HC with protein A-Sepharose and analysed on a gradient gel (4–20%) under non-reducing conditions with a single reducing lane as indicated (red). (**E**) The LC and HC compositions of the products in the medium were analysed by 2D gel electrophoresis with the first non-reducing dimension and a second reducing dimension as indicated. Samples were prepared by pulse labelling cells and chasing for 3 h. The positions of the reduced HCs and LCs in the second dimension are as indicated. (**F**) An identical experiment as in **E** was carried out with the 277 HC construct to illustrate the formation of HC dimers secreted into the medium.

The assembly of an antibody molecule is initiated by the formation of a HC dimer as a consequence of an interaction between the C_H_3 domains [25]. The dimers formed are then stabilised by the formation of an interchain disulphide within the hinge region between the C_H_1 and C_H_2 domains. Two LCs then associate with the HC dimer to form a tetramer, which is stabilised by further interchain disulphides between the H and L chains [3]. To illustrate the various intermediates formed during this pathway, we transiently co-expressed HC and LC coding for a humanised mouse monoclonal antibody (A33) [13] in either an IgG1 or IgG4 format. The IgG4 construct used for these experiments contains a mutation within the hinge region (S225P), which has been shown to reduce the heterogeneity of secreted IgG4 intermediates [26], specifically HC : LC dimers [20]. The HC-coding region consists of human C_H_1, C_H_2, C_H_3 and mouse V_H_ domains, whereas the LC constructs consist of human C_L_ and mouse V_L_ domains. Western blot analysis of the intracellular material using a fluorescent protein A illustrated three prominent products, previously characterised as fully assembled IgG, HC dimers and some trimers consisting of one LC and two HCs [20] (Figure 1A,B). Only fully assembled IgG or HC dimers were observed in the secreted material — note that protein A does not bind to all intermediates such as LC dimers. Interestingly, the secreted HC dimer had a faster mobility than the non-secreted dimer, indicating the presence of intrachain disulphide(s) that alter the hydrodynamic volume of the denatured protein and subsequently their electrophoretic mobility. As the disulphides within each of the separate HC domains apart from the C_H_1 domain form prior to the dimerisation event, it is likely that this shift in mobility indicates the formation of the C_H_1 intrachain disulphide.

The secretion of HC dimers was further confirmed by pulse labelling cells co-transfected with the IgG4 HC and LC followed by a chase for up to 5 h (Figure 1C,D). Products were immunoisolated with antibodies to the LC and HC. Fully assembled and interchain disulphide bonded IgG4 as well as several intermediates were secreted into the medium during the 5 h. Each intermediate was characterised for the presence of HC with or without a LC by carrying out non-reducing/reducing two-dimensional electrophoresis (Figure 1E). The product migrating at ∼100 kDa contained only HCs confirming its identity as an HC dimer. In addition, we carried out a similar experiment with a different HC construct (277), which has a variable region different from A33 (Figure 1F). The presence of only HC in the product migrating at 100 kDa indicates that the formation of HC dimers is not restricted to the A33 construct.

**Figure 2.**
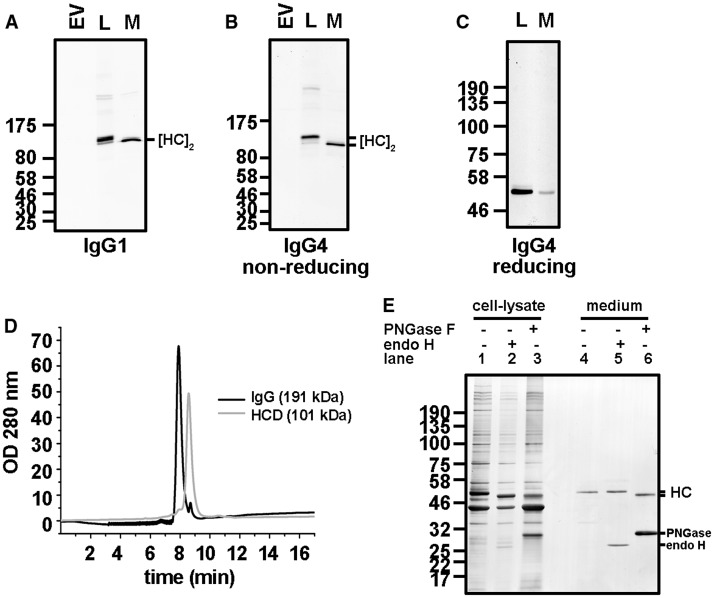
LC-independent HC dimer secretion. CHO-S cells were transfected with expression constructs coding for the HC IgG1 (**A**), HC IgG4 (**B–E**) or co-transfected with the LC and HC IgG4 (**D**) of A33. Cells transfected with the empty vector are as indicated (EV). (**A** and **B**) Immunoblot analysis of the intracellular (L) and secreted (M) HC products separated on a 10% polyacrylamide gel under non-reducing conditions. Fluorescent protein A was used to detect antibody products containing the Fc dimer of the HC. The mobilities of the HC dimers are as indicated [HC]_2_. (**C**) Immunoblot analysis of the intracellular (L) and secreted (M) HC products separated on a 10% polyacrylamide gel under reducing conditions. Anti-IgG HC was used as a primary antibody and a fluorescent protein A as a secondary for detection. (**D**) Elution profile from SE chromatography of secreted products purified using protein A-Sepharose following co-transfection of an LC and HC (black line) or transfection with an HC alone (grey line). Approximate molecular mass of major species are as indicated. (**E**) Silver stain analysis of affinity-purified HC from the lysate (lanes 1–3) or medium (lanes 4–6) after digestion with endoH and PNGase F analysed on a 12.5% reducing polyacrylamide gel.

The presence of HC dimers stabilised by interchain disulphides in the secreted material could indicate a lack of assembly with the LC or simply that the LC–HC interchain disulphides had not formed. If the latter were the case, then non-covalent interactions between the HC and the LC could be sufficient to allow folding of the C_H_1 domain and release from ER retention. To test these possibilities, we first expressed HC constructs in the absence of a LC and determined whether the HC dimers formed were secreted. For both the A33 IgG1 and IgG4 constructs, we identified HC dimers stabilised by disulphides in the culture medium indicating secretion (Figure 2A,B). There was a difference in mobility of the secreted HC dimers compared with the intracellular material, indicating the formation of intrachain disulphides in the secreted protein. The secreted and intracellular HC had the same mobility when separated under reducing conditions (Figure 2C). We also affinity-purified the secreted material, from either HC/LC co-transfections or transfections with HC alone, with protein A-Sepharose and separated the purified protein by SE chromatography carried out under native conditions (Figure 2D). As expected, the predominant species from the co-transfection was fully assembled IgG with an elution time indicative of a 191 kDa tetramer. A second peak was observed with an elution time indicative of a 101 kDa HC dimer. The purified protein from the HC-only transfections had an identical elution time to this second peak. These results confirm that A33 HC dimers are secreted either when HCs are expressed alone or when co-transfected with a LC, indicating that their presence in the denatured samples was not due to a lack of interchain disulphides.

The presence of HC dimers in the cell culture medium might also be a consequence of cell lysis, releasing the otherwise retained intracellular protein. To evaluate this possibility, we affinity-isolated intracellular and secreted proteins using protein A-Sepharose and then subjected the resulting samples to PNGase or endoH digestion. The proteins were then separated by SDS–PAGE and visualised by silver staining (Figure 2E). As the HC is glycosylated, treatment with endoH should result in cleavage of the oligosaccharide side chain with a resulting increase in electrophoretic mobility if the protein had been retained in the ER. The passage of glycoproteins through the Golgi apparatus results in modification of the oligosaccharide such that it becomes resistant to endoH digestion [27]. Hence, endoH resistance demonstrates passage of the protein through the secretory pathway. PNGase cleaves all oligosaccharide side chains irrespective of whether the glycoprotein has passed through the Golgi or not. The isolated intracellular HC was as expected sensitive to digestion with both endoH and PNGase (lanes 1–3). However, the HC in the medium was resistant to digestion with endoH and sensitive to PNGase. These results confirm that the HC in the medium has passed through the secretory pathway and is not present as a result of cell lysis. Hence, we have shown that, for this particular humanised antibody, there is a lack of the normal quality control mechanism that should prevent the secretion of HC dimers.

### The LC-independent secretion of HCs is variable region-dependent

**Figure 3.**
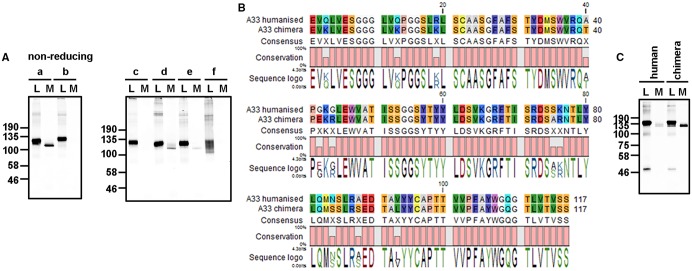
LC-independent HC dimer secretion is dependent of the VH domain of the HC. CHO-S cells were transfected with expression constructs coding for the HC IgG4 containing differing V_H_ domains. (**A**) Immunoblot analysis of the intracellular (L) and secreted (M) HC products separated on a 7.5% polyacrylamide gel under non-reducing conditions. Fluorescent protein A was used to detect antibody products containing the Fc dimer of the HC. The HCs used included an in-house antibody (277) (a) and constructs with variable domains that have specificity for TNF-α (b), CD20 (c), CD25 (Zenapax) (d) CD25 (Simulect) (e) or CD11a (f). (**B**) Sequence comparison of the mouse and humanised V_H_ domain sequence highlighting the 11 amino acid differences. (**C**) Immunoblot analysis of intracellular (L) and secreted (M) HC dimers expressed following transfection with the humanised or chimeric A33 HC. Analysis was under non-reducing conditions on a 7.5% polyacrylamide gel using a fluorescent protein A for detection.

To determine whether other engineered HCs can be secreted in the absence of an LC, we created a series of constructs containing the human IgG4 constant domains each with different variable domains. Each HC was transfected individually into CHO-S cells, and the cell lysate or medium was probed for the presence of HC dimers by western blotting (Figure 3A). The variable domains code for antibodies raised in mice that either have not been altered (c and e) or have a varying number of different point mutations to humanise the mouse variable domain (a, b, d and f). Expression of HC a, d and e resulted in some HC dimer secretion, with HC b, c and f dimers being efficiently retained in the cell. Each of the secreted HC dimers had a faster mobility than the cellular protein, indicating that some additional intrachain disulphide bond(s) had formed. The levels of expression of each of the HCs were equivalent, suggesting that the differential secretion of the HC dimers was not due to saturation of the ER quality control system. These results show that the variable domain determines the extent of HC dimer secretion.

To evaluate further the role of the variable region in HC dimer secretion, we assessed an additional A33 construct that had been through a process of further engineering to humanise the mouse variable domain. The sequence contains 11 amino acid changes compared with the original mouse sequence (Figure 3B). These changes led to a reduced level of secretion of HC dimers (Figure 3C), but did not prevent secretion. Taken together, the results show that the variable domain sequence influences the level of LC-independent HC secretion and that subtle changes can influence the extent of secretion.

### Folding of the C_H_1 domain in the absence of LC

**Figure 4.**
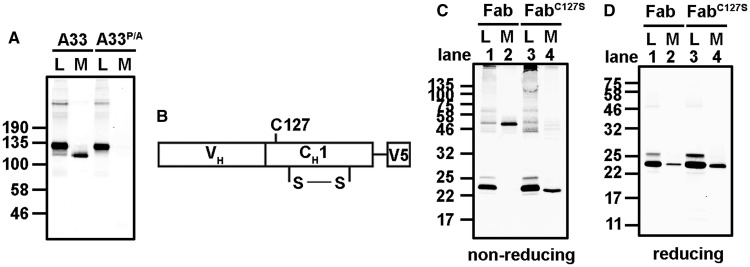
Dimerisation of the A33 HC does not require the C_H_3 domain but does require folding of the C_H_1 domain. CHO-S cells were transfected with expression constructs coding for the HC IgG4 of A33 and C_H_1 mutant P151A (**A**), the Fab HC IgG4 of A33 and a C_H_1 mutant C127S (**C** and **D**). (**A**) Immunoblot analysis of the intracellular (L) and secreted (M) products separated on a 7.5% polyacrylamide gel. Fluorescent protein A was used to detect HC products containing the Fc dimer. (**B**) Schematic representation of the Fab HC construct highlighting the intramolecular disulphide bond and the free cysteine which would normally form an intermolecular disulphide bond with the C_L_ domain. (**C** and **D**) Immunoblot analysis of Fab HC (lanes 1–2) and Fab HC C127S mutant (lanes 3–4) under non-reducing and reducing conditions separated on a 12.5% polyacrylamide gel. Detection of immunoblots was with an anti-V5 primary antibody and fluorescent protein A.

Previous results and those presented here would suggest that the variable domain can influence C_H_1 domain folding either negatively, in the case of antibodies selected during B-cell development, or positively, following antibody engineering during the humanisation process. The formation of an intrachain disulphide within the C_H_1 domain would suggest that the C_H_1 domain has folded in the secreted HC dimers. To determine whether preventing C_H_1 folding would restore the retention of the A33 HC by the cellular quality control system, we mutated a proline within this domain which has previously been shown to be required for correct folding [7]. In contrast with the wild-type protein, the P151A mutant was efficiently retained within the cell when transfected alone (Figure 4A). HC dimers were still able to form as evidenced by the mobility of the intracellular protein separated under non-reducing conditions. However, no intrachain disulphide formation was evident in the intracellular material. These results suggest that, in the absence of C_H_1 domain folding, the A33 HC is efficiently retained in the cell. The fact that we see secretion of wild-type A33 HC dimers suggests that the subversion of ER retention is due to folding of the C_H_1 domain.

### Variable domain-dependent folding of the C_H_1 domain can occur with Fab fragments

The C_H_3 domain initiates the assembly of the HC dimers which form prior to C_H_1 folding and assembly of the [HC]_2_[LC]_2_ tetramer [3]. To determine if the C_H_3-mediated dimerisation event is required for the secretion of the A33 HCs, we made a HC-Fab construct (V_H_ and C_H_1 domains without the hinge region) containing a V5-epitope tag (Figure 4B). Following transfection, the predominant species present within the cell lysate was found to be the Fab HC monomer (Figure 4C,D, lane 1). Fab HC dimers were present in the medium, demonstrating the secretion of Fab HCs in the absence of a LC (Figure 4C, lane 2). These dimers were stabilised by the formation of an interchain disulphide as evidenced by the difference in electrophoretic mobility of the proteins separated under reducing or non-reducing conditions. This result demonstrates that the A33 HC-Fab chains can form dimers in the absence of the C_H_3 domain. To determine whether the interchain disulphide was formed between the C_H_1 domains, we mutated the serine to cysteine (C127) that normally forms a disulphide between the C_H_1 and the C_L_ domains. This mutation prevented the formation of an interchain disulphide bonded dimer but did not prevent secretion of the Fab HC (Figure 4C,D, lane 4). The formation of the interchain disulphide in the secreted Fab HCs via the C_H_1 domain cysteines indicates that the folded C_H_1 domains interact directly within the dimer. Taken together, our results demonstrate that the variable domain can influence the folding of the C_H_1 domain, so that it can fold in the absence of its cognate LC.

## Discussion

Mammalian cells have evolved elegant mechanisms to ensure that only correctly folded proteins are transported from the ER to later stages of the secretory pathway [28]. One such mechanism ensures that only assembled antibody molecules are secreted and involves the retention of HC dimer intermediates within the ER by the interaction of an unfolded C_H_1 domain with the ER-resident protein BiP [5]. Here, we demonstrate that this mechanism is subverted when some HCs with engineered variable regions are expressed. The results highlight that variable domains that can subvert the ER quality control process are selected against in B-cell development during the pro- to pre-B-cell transition [12]. How the variable domain is able to influence the ER quality control system is yet to be established, but our results suggest that stabilisation of the C_H_1 domain must be involved. Folding of the C_H_1 domain can occur in these HCs without a LC, suggesting that interactions with the folded V_H_ domain facilitate C_H_1 folding and the formation of its intrachain disulphide.

Understanding the characteristics of the variable regions that stabilise the C_H_1 domain could influence the selection of variable domains during the humanisation process and the development of antibody therapeutics. However, the number of variable domains analysed in the present study is too small to identify any such characteristics. There was a lessening of the level of LC-independent secretion of HC upon humanisation of the A33 variable domain, but we also saw efficient retention of a HC construct which has an unaltered mouse sequence (HC (c) in Figure 3A). Previously, a pool of 18 immunoglobulin µ HCs that were isolated from pro-B-cells were secreted from COS7 cells in the absence of a LC [12]. Sequence analysis of the variable domains revealed few common features that were different from the HCs that are dependent on a LC for their secretion. One characteristic that was prevalent among domains was an enrichment of positively charged amino acid side chains in the CDR3 region. However, this was not a common feature of all the HC pool. Indeed, there is no preference for such positively charged side chains in the CDR3 region of the variable domains that we find can cause HC dimer secretion (data not shown). Resolution of this issue will require a much larger pool size or structural details of the secreted HC dimers illuminating the interaction interface between the V_H_ and C_H_1 domains.

The ability of the HC-Fab construct to form dimers and be secreted indicates an intrinsic ability of the V_H_ or C_H_1 domains to interact, even in the absence of C_H_3-mediated dimerisation. It is known that LCs also can form dimers that can be secreted in the absence of an HC [29]. Export competence is variable region-dependent, arguing for an initial association of the V_L_ domains with a subsequent formation of an interchain disulphide and secretion. The interchain disulphide forms between carboxyl-terminal cysteine residues that would normally form a disulphide between the LC and HC. In the case of the HC-Fab construct, we show that the dimers ultimately associate via their C_H_1 domains as an interchain disulphide forms between cysteines located within this domain. This does not preclude an initial association of the V_H_ domain. When the A33 HC is expressed in the presence of a LC, the most abundant species secreted is the fully assembled antibody tetramer. This suggests that, while HC dimers can form via association between the V_H_ and C_H_1 domains, the preferred interaction is with the LC.

Our results go some way to explain heterogeneity in assembly and secretion of authentic and novel antibody formats. Altering the combination and order of variable and constant domains is highly likely to have an impact on the propensity for stabilisation of the C_H_1 domain, resulting in subversion of the ER quality control system. An evaluation of the consequence of novel antibody format on the stringency of ER retention of unassembled intermediates would be an appropriate step as part of the design process. In addition, selecting against HCs that demonstrate LC-independent secretion as part of the humanisation process would be a valuable empirical approach to increase antibody yields and reduce the heterogeneity of the secreted product.

## Abbreviations

BiP: binding protein
CHO: Chinese hamster ovary
DMEM: Dulbecco's Modified Eagle Medium
DTT: dithiothrietol
ER: endoplasmic reticulum
HCs: heavy chains
LCs: light chains
NEM: *N*-ethyl maleimide
PEI: polyethylenimine
SE: size exclusion
